# Altered cortical beta‐band oscillations reflect motor system degeneration in amyotrophic lateral sclerosis

**DOI:** 10.1002/hbm.23357

**Published:** 2016-09-13

**Authors:** Malcolm Proudfoot, Gustavo Rohenkohl, Andrew Quinn, Giles L. Colclough, Joanne Wuu, Kevin Talbot, Mark W. Woolrich, Michael Benatar, Anna C. Nobre, Martin R. Turner

**Affiliations:** ^1^ Nuffield Department of Clinical Neurosciences University of Oxford United Kingdom; ^2^ Oxford Centre for Human Brain Activity, Department of Psychiatry University of Oxford United Kingdom; ^3^ Department of Neurology, Miller School of Medicine University of Miami Florida

**Keywords:** motor neurone disease, magnetoencephalography, neurophysiology, neuroimaging, inhibition, biomarker

## Abstract

Continuous rhythmic neuronal oscillations underpin local and regional cortical communication. The impact of the motor system neurodegenerative syndrome amyotrophic lateral sclerosis (ALS) on the neuronal oscillations subserving movement might therefore serve as a sensitive marker of disease activity. Movement preparation and execution are consistently associated with modulations to neuronal oscillation beta (15–30 Hz) power. Cortical beta‐band oscillations were measured using magnetoencephalography (MEG) during preparation for, execution, and completion of a visually cued, lateralized motor task that included movement inhibition trials. Eleven “classical” ALS patients, 9 with the primary lateral sclerosis (PLS) phenotype, and 12 asymptomatic carriers of ALS‐associated gene mutations were compared with age‐similar healthy control groups. Augmented beta desynchronization was observed in both contra‐ and ipsilateral motor cortices of ALS patients during motor preparation. Movement execution coincided with excess beta desynchronization in asymptomatic mutation carriers. Movement completion was followed by a slowed rebound of beta power in all symptomatic patients, further reflected in delayed hemispheric lateralization for beta rebound in the PLS group. This may correspond to the particular involvement of interhemispheric fibers of the corpus callosum previously demonstrated in diffusion tensor imaging studies. We conclude that the ALS spectrum is characterized by intensified cortical beta desynchronization followed by delayed rebound, concordant with a broader concept of cortical hyperexcitability, possibly through loss of inhibitory interneuronal influences. MEG may potentially detect cortical dysfunction prior to the development of overt symptoms, and thus be able to contribute to the assessment of future neuroprotective strategies. *Hum Brain Mapp 38:237–254, 2017*. © **2016 Wiley Periodicals, Inc.**

## INTRODUCTION

The neurodegenerative disorder amyotrophic lateral sclerosis (ALS) is characterized clinically by progressive motor neuronal system degeneration from cortex to muscle. It is now understood to have multiple aetiologies [Turner and Swash, 2015] and shares clinical, pathological, and genetic overlap with frontotemporal dementia (FTD) [Phukan et al., 2012]. The term primary lateral sclerosis (PLS) encompasses a very slowly progressive phenotype with pure upper motor neuron (UMN) degeneration [Pringle et al., 1992]. The improved characterization of the genetic substrate for familial ALS now enables the study of asymptomatic mutation carriers predisposed to developing ALS [Benatar et al., 2013]. Identification of the earliest pathological events might inform future therapeutic efforts to slow disease progression prior to irreversible neuronal injury.

Neuroimaging has been at the forefront of the drive to explore in vivo cerebral pathology in ALS, concurrently developing candidate biomarkers [Turner and Verstraete, 2015]. Abnormal cortical functional connectivity, typically appraised by coherent fluctuations in the task‐free blood oxygen level‐dependent (BOLD) functional MRI signal, is linked to structural cerebral pathology in ALS [Douaud et al., 2011; Schmidt et al., 2014]. Reflecting the pathological overlap between ALS and FTD, some network specific connectivity changes are shared across these highly related diseases [Trojsi et al., 2015]. Abnormal increased functional connectivity has been demonstrated within specific cortical subregions [Agosta et al., 2013; Douaud et al., 2011; Zhou et al., 2014], perhaps underpinned by cortical hyperexcitability. This potentially pathogenic mechanism is also implicated by abnormal responses to transcranial magnetic stimulation (TMS) [Vucic et al., 2013b], which may reflect loss of cortical inhibitory interneuronal influences [Turner and Kiernan, 2012; Turner et al., 2005a, 2005b].

Direct noninvasive recording of cortical neurophysiology supplements existing functional MRI findings by harnessing millisecond temporal precision at the expense of reduced spatial resolution. The cortical neuronal dynamics underlying motor performance can be ascertained with particularly high sensitivity using magnetoencephalography (MEG) [Proudfoot et al., 2014a, 2014b]. Movement preparation and execution are consistently associated with modulations to motor and premotor neuronal oscillation power, particularly within the beta‐band (15–30 Hz) [Pfurtscheller and Lopes Da Silva, 1999]. Beta‐band limited power is initially reduced (alternatively described as desynchronization) but after movement termination a relative increase (or synchronization) in power follows, accompanied by fluctuation in corticospinal excitability [Chen et al., 1998; Fry et al., 2016; Kilavik et al., 2013]. MEG allows neural signal analysis from anatomically precise cortical structures. As well as their potential as biomarkers, such signals are of heightened relevance in ALS given their proposed use as a user input to brain‐computer interfaces in the advanced stages of disability [Grosse‐Wentrup and Schölkopf, 2014; Kasahara et al., 2012].

Two electroencephalographic (EEG) studies directly considered the effect of ALS on these important neurophysiological markers of cortical activation. Reduction in cortical postmovement synchronization (termed postmovement beta rebound, PMBR) was identified in both studies, but conflicting conclusions drawn regarding the integrity of peri‐movement event‐related desynchronization (ERD) [Bizovičar et al., 2014; Riva et al., 2012]. This study applied the combined temporal and spatial resolution uniquely afforded by MEG to a group of both typical ALS and PLS patients. The central hypothesis was that alterations to cortical beta‐band oscillations may reflect pathological cortical hyperexcitability, which is an early and distinguishing feature of ALS and related phenotypes (Geevasinga et al., 2015a, 2015b]. A group of asymptomatic ALS gene mutation carriers (AGCs) was included to appraise the sensitivity of MEG in the detection of cortical pathology prior to the development of overt symptoms.

## METHODS

### Participants

Apparently sporadic ALS and PLS patients (i.e., without a family history of ALS or FTD), both prevalent and incident cases, were recruited from a tertiary referral clinic as a component of the Oxford Study for Biomarkers in Motor Neurone Disease (“BioMOx”). Diagnosis was confirmed by one of two experienced neurologists (MRT, KT) according to consensus criteria [Brooks et al., 2000; Gordon et al., 2006]. Six of the ALS patients were taking Riluzole at the time of study. Healthy controls, typically spouses of patients, were similar in age, handedness, and level of education. AGCs included were recruited locally, and through collaboration with the presymptomatic Familial ALS (Pre‐FALS) study (MB, JW) [Benatar and Wuu, 2012], participants in which travelled to Oxford for both MEG and MRI. Demographics for all participant groups are detailed in Table I.

**Table 1:** Demographic and clinical data

Mean ± SD (range)	**ALS** (*n* = 11)	**PLS** (*n* = 9)	**Asymptomatic genetic carriers** (*n* = 12)	**Controls old** (*n* = 10)	**Controls young** (*n* = 10)
**Age** (years)	63.5 ± 7.6 (48:74)	59.6 ± 8.0 (44:70)	51.7 ± 9.9 (36:66)	61.7 ± 9.3 (45:75)	51.0 ± 9.5 (37:64)
**Gender**	9 M: 2 F	2 M: 7 F	2 M: 10 F	4 M: 6 F	3 M: 7 F
**Handedness**	11 R	9 R	12 R	9 R: 1 L	8 R: 2 L
**Site of onset** or **genetics**	1 bulbar	1 bulbar	10 *SOD1*	N/A	N/A
	2 respiratory	2 both legs	2 *C9orf72*		
	1 RUL, 3 LUL	1 RLL, 5 LLL			
	2 RLL, 2 LLL				
**ALSFRS‐r**	34.8 ± 8.8 (21:48)	35.1 ± 6.3 (24:43)	N/A	N/A	N/A
**Disease Duration** from Symptom Onset (months)	23.7 ± 18.9 (5:72)	121.0 ± 57.2 (47:283)	N/A	N/A	N/A
**Progression Rate** (48 – ALSFRS‐R/duration in months)	0.79 ± 0.69 (0:2.4)	0.12 ± 0.06 (0.05:0.46)	N/A	N/A	N/A
**Cognition** score (% correct) Intact/Borderline/Impaired	84.8% ± 8.5 (69:95)	84.6% ± 9.4 (73:95)	91% ± 4.5 (80:97)	96 .1% ± 3.1 (91:100)	96.3% ± 2.4 (92:99)
	(10 ECAS, 2 ACE‐r) 8/1/2	(8 ECAS, 1 ACE‐r) 5/1/3	(11 ACE‐r)	(9 ACE‐r)	(8 ACE‐r)

Mean followed by SD (range). Genetic data only available for AGCs.

Clinical and cognitive assessments (MRT and MP) were performed on the same day as MEG acquisition (MRT, GR, and MP). Contemporaneous T1‐weighted structural MRI scans were acquired for coregistration with the MEG data (3T Siemens Trio, MPRAGE sequence). Three ALS patients were unable to tolerate MRI, and a standard Montreal Neurological Institute (MNI) template was instead used for MEG coregistration. An additional healthy control was excluded due to incidental white matter changes. Disability was assessed using the revised ALS Functional Rating Score (ALSFRS‐R, range 0–48, lower scores reflecting greater disability). Cognitive function was predominantly assessed using the Edinburgh Cognitive and Behavioral ALS Screen (ECAS) [Abrahams et al., 2013], or the revised Addenbrooke's Cognitive Examination (ACE‐R) [Mioshi et al., 2006] for those cases studied prior to development of the ECAS. Rate of disability progression (ΔALSFRS‐R) was calculated as the decrease in ALSFRS‐R from a presumed baseline score of 48, divided by the disease duration in months from reported symptom onset. A measure of the burden of clinical UMN signs was based upon a pathological reflex sum score [Menke et al., 2014; Turner et al., 2004]

All participants provided written informed consent. The study was approved by the National Research Ethics Service South Central Oxford Research Ethics Committee B (08/H0605/85), and South Central Berkshire Committee (14/SC/0083).

### Task Design

**Figure 1:**
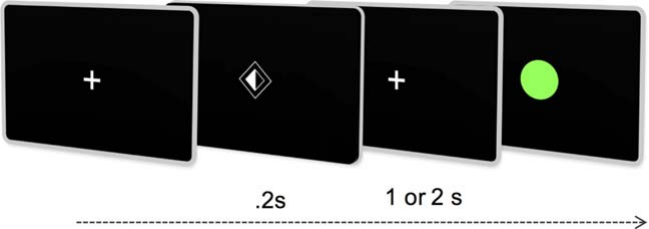
Task design schematic demonstrating visual cues instructing lateralized motor preparation for 1 or 2 s (dependent on cue style) followed by response only to green Go targets. [Color figure can be viewed at http://wileyonlinelibrary.com.]

A Go‐NoGo task was designed (ACN and GR) to investigate neural activity related to spatially selective motor preparation. Monochromatic visual cues, with one side shaded indicating the hand to be moved, were presented foveally for 200 ms, followed by an interstimulus interval (ISI) of either 1 or 2 s, during which a central fixation cross remained. After the variable time period of lateralized motor preparation, the fixation cross was replaced by either a green “Go” circular target in 80% of trials, or a red “NoGo” target in 20% (randomly distributed; Fig. 1). Participants were instructed to make rapid responses to just the “Go” targets by lifting and replacing the index finger of only the prepared hand. Trials were randomly distributed in laterality and ISI duration. A maximum of three blocks of 100 trials (median intertrial interval 6.25 s) were acquired per participant. Fatigue limited acquisition to two blocks in one PLS patient. Each block lasted ∼12 min. Stimuli were created on Matlab and presented via the Psychtoolbox package [Brainard, 1997].

### MEG Data Acquisition

Behavioral responses were measured using a fiber‐optic sensing device, and confirmed by off‐line inspection of surface electromyography (EMG) traces recorded from the extensor digitorum communis. To ensure that participants were engaged in motor preparation, only those trials in which correct responses were confirmed were included in subsequent analyses. Visual fixation and blinks were monitored using an infrared eyetracker (Eyelink 1000) as well as vertical and horizontal EOGs, and subsequently checked off‐line.

MEG data were acquired at the Oxford Centre for Human Brain Activity (OHBA) on a passively shielded Elekta Neuromag system comprising 204 orthogonally oriented planar gradiometers and 102 magnetometers. Participants were seated comfortably, 90 cm away from a back‐projected screen (Panasonic PT D77OOE). Continuous adjustment was made for head position within the MEG helmet using four emitting coils secured to the participant's scalp. A Polhemus 3D tracking system recorded coil positions relative to nasion and preauricular fiducial landmarks, alongside distributed points covering the scalp surface. MEG data were digitized at 1000 Hz with a 0.03 Hz highpass filter.

### MEG Data Preprocessing

A locally developed analysis pipeline, OHBA Software Library (MW), was used for analysis, incorporating Matlab toolboxes from SPM12 (UCL, London, UK), FSL (FMRIB, Oxford, UK), and FieldTrip (Donders, Nijmegen, NL). Continuous MEG data were initially inspected to identify noise‐corrupted channels. Maxfilter software (Version 2.2, Elekta) was then applied to the remaining channels for Signal Source Separation and head position adjustment, transforming the data into a set of virtual sensors. Data were then downsampled to 250 Hz, and physiological artefacts were identified and removed using an independent component analysis classification tool based on visual inspection of component spatial topography and timecourse [Baker et al., 2014]. Exactly two independent components pertaining to (1) cardiac pulse and (2) eye‐blink were removed from each data set. Data were then epoched according to the time‐period of interest (including a one second pre‐cue baseline period) for subsequent analysis. Alongside behaviorally incorrect trials and those with premature responses identified by surface EMG, epochs contaminated by artefact were identified by an automated rejection tool, and removed from the analysis. This comprised 1.3% of trials, on average, over subjects. The tool used a robust bisquare linear regression to fit for the mean standard deviation in the data over trials. Any trials that were down weighted by more than 99% points during the regression were classed as outliers. Epochs were centred on either (1) the preparatory cue, to investigate neural correlates of motor preparation, (2) response time (RT), to investigate motor execution while accounting for differences in reaction time, (3) movement termination time, to investigate PMBR, or (4) target appearance, to investigate successfully withheld “NoGo” trials.

### Source Analysis

A linearly constrained minimum variance scalar beamformer [Robinson and Vrba, 1999; Van Veen et al., 1997] was used via a single‐shell forward model [Sarvas, 1987] to project preprocessed MEG data, bandpass filtered between 15 and 30 Hz, onto a regular 3D grid spanning the entire brain. Estimates of the data covariance matrix were regularized by removing the weakest PCA components [Woolrich et al., 2011] to leave only 61 (the approximate rank of the data after the use of Maxfilter and removal of two ICA components pertaining to (a) ECG and (b) eye‐movement artefacts). Cue, response and movement termination locked epochs were then inspected across 8‐mm whole‐brain grids to locate subject specific, functionally defined regions of interest (ROIs) corresponding to cortical motor regions: for each subject, the MNI coordinates separately pertaining to either the maximal preparatory ERD, response ERD or PMBR were selected for each hemisphere (locations depicted in Supporting Information Figure S1). Neural signals from these pairs of voxels were carried forward from each subject for comparison of timecourse dynamics. Note that an alternative analysis using the first principal component of the signals from the surrounding voxels within an 8 mm radius, yielded similar results (data not shown).

Time‐frequency transformations as implemented in the FieldTrip toolbox (http://www.fieltriptoolbox.org) were then applied. Hanning tapers of length 300 ms at 50 ms intervals within a frequency range 4–45 Hz were used to reveal the spectral distribution of ERD/S, and multitapers centered at 21.5 Hz with 8.5‐Hz frequency smoothing were used to display the timecourse of beta‐band power. Summary values for preparatory beta ERD (baselined mean value over 0–1.2 s post‐cue), response ERD (±200 ms around response), baselined PMBR (0.15–1.5 s from response completion) were generated per participant from the respective ERD or PMBR ROI data for subsequent clinical correlation. Cluster‐based permutation statistics as implemented in FieldTrip [Maris and Oostenveld, 2007] were used to compare the main task effects between participant groups. For exploration of non‐normal outcome measures, Kendall's Tau was chosen to assess the (uncorrected) 2‐tailed significance of correlations (SPSS, IBM).

Whole‐brain source‐space data (beta‐band, 15–30 Hz) were also compared across groups using a mass univariate trial‐wise general linear model (GLM) approach [Hunt et al., 2012; Woolrich et al., 2009]. The trial‐wise GLM consisted of different regressors that picked out the trials that corresponded to left and right lateralization, and “NoGo” and “Go” conditions. First‐level (within‐subject) contrasts of parameter estimates (COPEs), were then calculated comparing either left or right lateralized effect against baseline, or successfully withheld “NoGo” effect either against baseline or against “Go” effect. The first‐level COPES were averaged over repeated sessions to compute subject‐level means, which were in turn passed into the group‐level subject‐wise GLM. Group‐level regressors were set to model the group means and group contrasts set to detect group differences using unpaired t‐tests. Prior to passing subject‐level COPEs to the group‐level analysis, they were transformed such that only left‐hand preparation/response source‐space COPEs were flipped around the medial surface (i.e., around the x‐axis in MNI coordinates). This enabled merging of both response lateralities into contra‐lateral and ipsi‐lateral hemispheres, relative to the effector limb. 4D spatiotemporal maps were averaged across selected time windows of interest, corresponding to the all‐subjects combined group's maximal ERD following appearance of cue/target, or corresponding to (individual trial specific) response onset/offset. The resulting 3D maps were compared between groups using cluster‐based permutation statistics (Randomize, FSL), with a pre‐defined cluster‐forming threshold (t‐stat = 2.4) and 10,000 permutations with 100‐mm of spatial smoothing applied to the variance of the COPE to improve the effective degrees of freedom. Given the strong a priori expectation that group differences would be prominent within the motor cortices, group comparison was restricted to an anatomically confined binary mask encompassing bilateral motor areas.

The extent to which PMBR was confined to the contra‐lateral motor cortex was investigated using three independent measures of “degree of lateralization” (DoL). Firstly, dynamic beta power timecourses from each subject's motor ROI (as defined by trough beta ERD and subsequently by peak PMBR) were contrasted as a percentage ((contra‐ipsi)/(contra + ipsi)] prior to group comparison over time. Secondly, a 3D voxel‐wise lateralization effect was calculated over the time‐averaged period of maximal PMBR across all subjects (1:2.5s post movement), and over a 400 ms window centered on each group's peak PMBR time‐point. The resultant subject specific DoL spatial map was contrasted between groups using cluster‐permutation tests as above.

Lastly, the correlation between fluctuations of beta power in the same PMBR time‐period were assessed. Using anatomical parcels (from the Harvard‐Oxford cortical structural atlas), time‐courses for each motor cortex were extracted by taking the first principal component over the enclosed voxels, using an orthogonalization procedure to correct for spatial leakage from surrounding anatomical parcels [Colclough et al., 2015]. Correlations were taken between the Hilbert power envelopes of these corrected signals and converted to normal variates with Fisher's transformation. These correlations were averaged within subjects and contrasted between groups, performing inference with 5000 permutations of the subject labels.

The expected oscillatory signature of response inhibition has been shown to involve an augmentation of beta power relative to unsuccessful inhibition [Huster et al., 2013]. The parcellation, orthogonalization and correlation method was additionally applied to an exploratory analysis of “NoGo” trial epochs, assessing the functional connectivity between the motor cortices and two anatomical regions commonly implicated in response inhibition: the supplementary motor area (SMA) and right inferior frontal cortex (rIFC) [Aron et al., 2014].

## RESULTS

### Demographic and Behavioral

Twelve ALS and 10 PLS patients underwent MEG acquisition. One ALS participant was excluded from analysis after failing to recognize the laterality of the cue. MEG source space variance maps for one PLS participant revealed heavy artefact contamination. Therefore MEG data from the remaining 11 ALS patients (9 male, mean age 63.5 ± 7.6 years) were contrasted against nine patients with PLS (2 male, 59.6 ± 8.0 years) and 10 age‐matched healthy controls (4 male, 61.7 ± 9.3 years). Twelve asymptomatic gene carriers (AGCs; 2 male, 51.7 ± 9.9 years, 10 *SOD1*, 2 *C9orf72*) were contrasted against another age‐matched selection of 10 healthy controls (3 male, 51.0 ± 9.5 years). Six healthy controls were included in both age comparators. Full details are given in Table 1.

**Figure 2:**
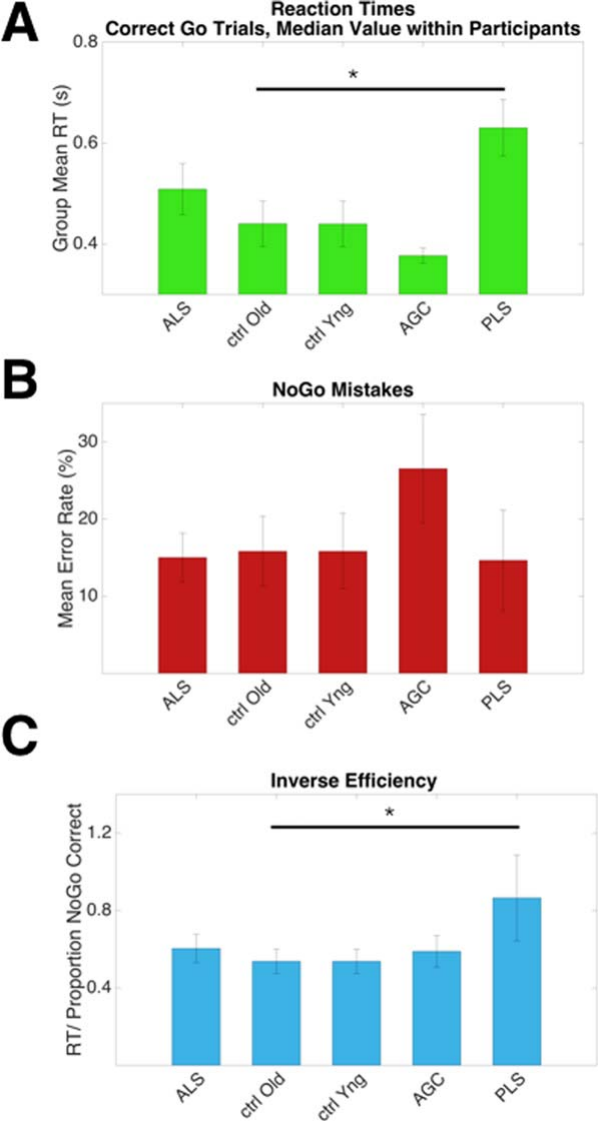
Behavioral data detailing task performance across groups. **A:** PLS patients responded slower than older controls, *P* = 0.016. **B:** A trend towards more NoGo errors was made by pre symptomatic carriers. **C:** Inverse efficiency, a global measure of task performance (RT/NoGo proportion correct) was impaired in the PLS group (*P* = 0.022). Median values within participants, mean values within group, error bars = SEM. Uncorrected for multiple comparisons. [Color figure can be viewed at http://wileyonlinelibrary.com.]

ALS patients tended to complete fewer correct “Go” responses than other groups; mean ± standard deviation 196 ± 66 from a maximum possible 240 (healthy controls 228 ± 18, *P* = 0.14). An effect of group membership was noted on RT, (*F*(4,42) = 5.44, *P* = 0.001, Fig. 2A), with PLS patients responding the slowest (623 ms, significantly slower than older controls, 440 ms, *P* = 0.016) and AGCs the fastest (377 ms, not significantly faster than younger controls, 434 ms, *P* = 0.17). Throughout the groups there was a significant effect of ISI duration on RT, such that participants were on average 35 ms faster following the longer ISI (*F*(1) = 19.0, *P* < 0.001). There was no group by ISI duration interaction.

The AGC group mean for erroneous responses on “NoGo”' trials was the highest at 26.5%, although not significantly more so than healthy controls (10.6%, *P* = 0.08, uncorrected, Fig. 2B). A global measure of task performance, inverse efficiency, was calculated as RT/NoGo” proportion correct. PLS patients performed worse relative to older controls (*P* = 0.022, uncorrected, Fig. 2C).

An increase in RT in the “Go” trials following a “NoGo” trial by 20 ms on average was consistently seen across all groups (Supporting Information Figure S2). This was an expected behavioral effect, equivalent to motor slowing induced by novel stimuli [Wessel and Aron, 2013].

### Motor Preparation

**Figure 3:**
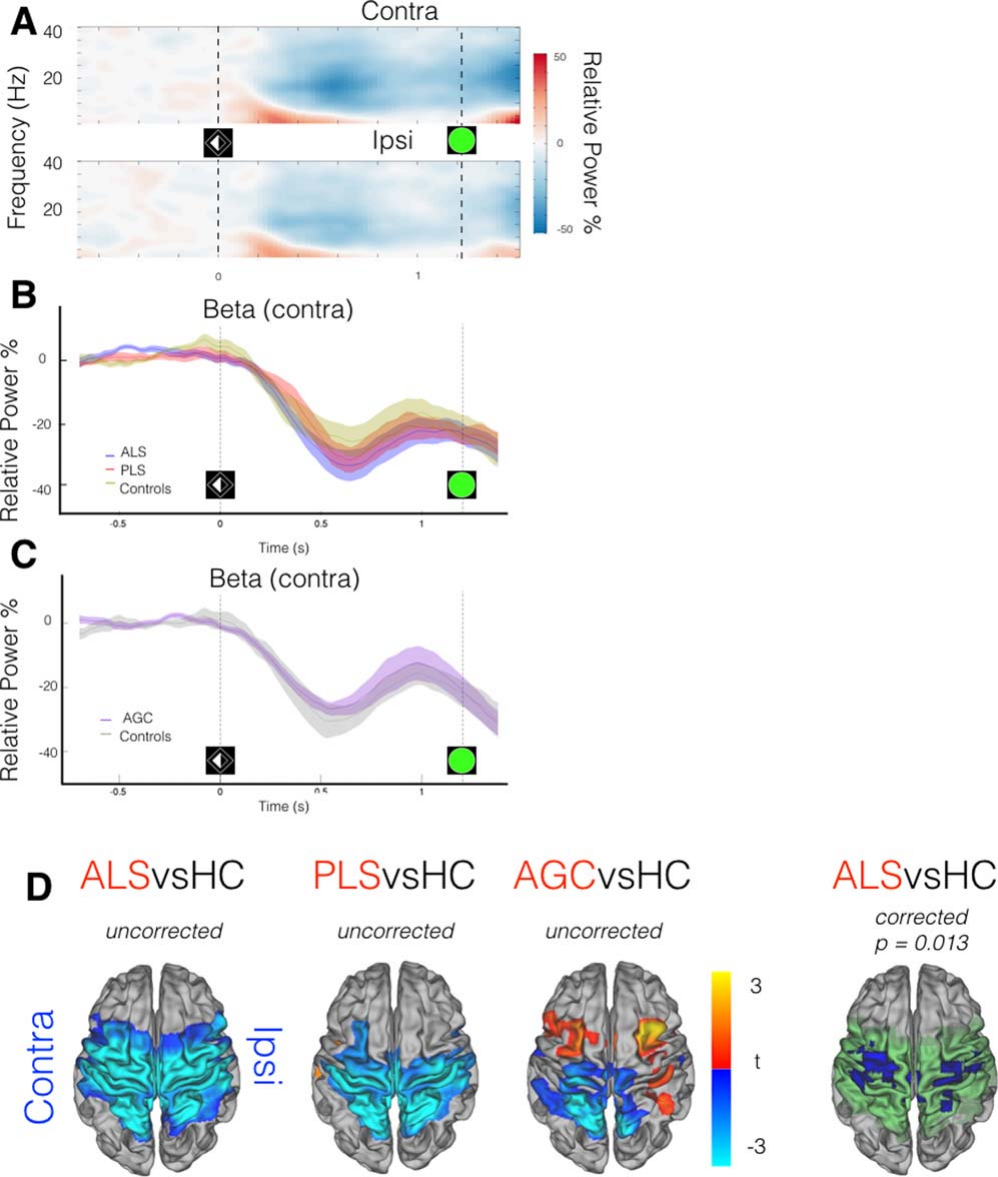
MEG data epoched around cue (*t* _0_ = laterality cue appearance). **A:** Neural correlate of task performance across frequency range demonstrated in TFR (averaged across healthy controls only) from motor cortex ROIs, contra/ipsilateral relative to effector limb. Beta‐band (15:30 Hz) power decrease (desynchronization) from baseline (more intense desynchronization = deeper blue) occurs during motor preparation, particularly in the contralateral hemisphere, maximally 600 ms post‐cue. **B,C:** Beta‐band power (relative to baseline) within contralateral motor cortex ROI, group comparisons. **D:** ALS patients show deeper beta desynchronization. *t*_0_ + 500 ms: *t*_0_ + 700 ms. t stats in red/blue, motor cortex mask used for statistics in green. Cluster correction within motor cortex mask, not across groups. Vertical lines denote appearance of visual laterality cue and go/Nogo target. [Color figure can be viewed at http://wileyonlinelibrary.com.]

Lateralized cortical changes in preparation for movement occurred shortly after cue processing in the form of regional beta‐band desynchronization. All groups demonstrated this preparatory beta‐desychronization, which was maximal at around 600 ms following cue onset (healthy control data from motor ROIs shown in Fig. 3A), and was more prominent in the motor cortex contralateral to the prepared limb. No significant group differences were immediately apparent in the intensity of the preparatory beta desychronization from subject‐specific functionally defined ROIs (Fig. 3B,C). However, analysis of whole‐brain MEG data, time‐averaged around the maximal beta desychronization (*t*_0_ + 500 ms: *t*_0_ + 700 ms. *t*_0_ = cue onset), revealed ALS patients to exhibit regions with significantly increased beta desychronization relative to controls, within (but not restricted to) both the contralateral and ipsilateral precentral gyrus (*P* = 0.031, Fig. 3D). Directionally similar trends were noted in both PLS and AGC groups.

### Motor Execution

**Figure 4:**
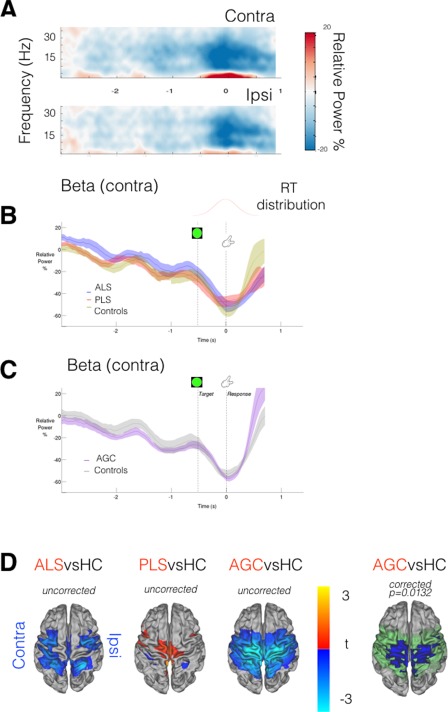
MEG data epoched around movement initiation ( *t* _0_ = response execution). **A:** TFR (averaged across healthy controls) demonstrates movement ERD (in blue) as a bilateral motor cortical event. **B,C:** Beta‐band power (relative to baseline) within contralateral motor cortex ROI; group comparison. Vertical lines indicate cue onset prior to median RT (distribution overlaid). **D:** AGCs show deeper beta desynchronization [*t*_0_: *t*_0_ + 200 ms] relative to controls, with a directionally similar trend in ALS patients. t stats in red/blue, motor cortex mask used for statistics in green. Vertical lines denote appearance of Go target and timing of response. [Color figure can be viewed at http://wileyonlinelibrary.com.]

As a unilateral limb movement was executed, beta desychronization became more focal within the motor cortices, yet also more bilateral [control time‐frequency representation (TFR)] (Fig. 4A). The timecourse of the peri‐response beta desychronization from the contralateral motor cortex ROI revealed it to be preserved in both ALS and PLS patient groups (Fig. 4B). The peri‐response beta desychronization extracted from the ROI appeared deeper within the AGC group although not significantly so (*P* = 0.590; Fig. 4C). Group contrasts were then statistically appraised in whole‐brain data (*t*_0_: *t*_0_ + 200 ms. *t*_0_ = response). Although ALS patients exhibited only a trend towards deeper beta‐desychronization, AGCs demonstrated regions with significantly deeper beta desychronization than controls over a large cortical area encompassing both motor cortices (*P* = 0.013, Fig. 4D). This result was qualitatively similar after exclusion of the two *C9orf72* AGCs. No significant differences were found between PLS patients and controls.

### Motor Rebound

**Figure 5:**
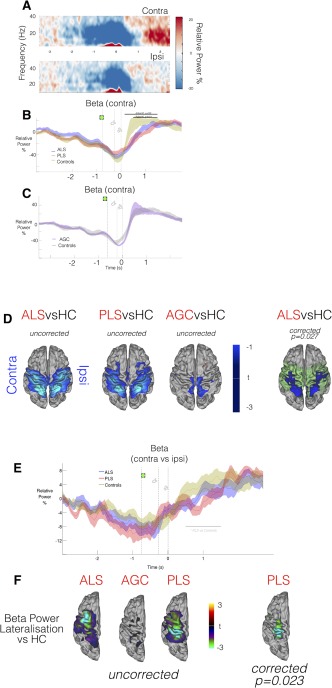
MEG data epoched around movement completion (*t* _0_ = finger replaced). **A:** TFR (averaged across healthy controls only) from motor cortex ROIs relative to effector limb. Post Movement Beta Rebound (PMBR, in red) is predominantly contralateral in healthy controls. **B,C:** Beta‐band power (relative to baseline) within contralateral motor cortex ROI; group comparison. Black bar = timespan of significant group difference via cluster permutation testing, *P* < 0.05. Vertical lines denote appearance of Go target, timing of response initiation and completion. **D:** ALS patients demonstrate lower beta power in early transition to PMBR [*t*_0_ + 500 ms: *t*_0_ + 700 ms]. t‐stats in blue, motor cortex mask used for statistics in green. **E:** Beta‐band power lateralization evolves over time around a response, PLS patients show a diminished DoL during PMBR (black bar, *P* < 0.05). **F:** PMBR DoL appraised in whole‐brain data [*t*_0_ + 1 s: *t*_0_ + 2.5 s]. PLS patients again show significantly reduced DoL. t‐stats in *ROY‐BIG‐BL*. [Color figure can be viewed at http://wileyonlinelibrary.com.]

Postmovement beta rebound was expected to be observed from 500 ms after termination of EMG activity, and typically lateralized to the contralateral precentral gyrus [Cheyne, 2013] (control group TFR Fig. 5A, other groups Supporting Information Figure S3, each individual displayed in Supporting Information Figure S4). Group differences in movement duration (*F* = 2.3, *P* = 0.07) were accommodated by again realigning data epochs (*t*_0_ = movement completion, as indicated by the fiber‐optic trigger). An expected correlation was found between RT and both EMG peak amplitudes (*P* < 0.001), and speed of transition in the contralateral motor cortex from “beta desychronization” (ERD) to PMBR (*P* = 0.0045) [Erbil and Ungan, 2007; Stancák and Pfurtscheller, 1996]. Inspection of beta power timecourses from the contralateral motor cortex confirmed significantly delayed beta rebound in both ALS (*P* = 0.03) and PLS (*P* = 0.011) patients (Fig. 5B).

AGCs showed a more rapid and increased amplitude beta rebound, though not significantly so (*P* = 0.062, Fig. 5C). Given this unexpected result in the AGC group, averaged rectified surface EMG timecourses were inspected, and confirmed faster EMG onset/offset relative to controls (Supporting Information Figure S5). Given that the transition from beta desychronization to beta rebound is a dynamic process, whole‐brain analysis was focused on the initial time‐period of beta rebound (*t*_0_ + 500 ms: *t*_0_ + 700 ms). During this period, ALS patients were confirmed to exhibit regions with significantly reduced beta power (*P* = 0.028) relative to controls (Fig. 5D), with a directionally similar trend in PLS patients.

Beta rebound in both patient groups was additionally suspected to be less lateralized, most marked in the PLS patients. This was confirmed only in beta power timecourses from the subject‐specific ERD defined motor ROIs (*P* = 0.017, Fig. 5E) but not in the PMBR defined ROIs. In the voxel‐wise spatial maps of DoL (Fig. 5F), a significant difference in lateralization was noted across a broad time‐period (1–2.5 s following movement completion) but group differences were not preserved in the 400 ms time‐period centered on each group's peak PMBR. This finding suggests a delay in PMBR, in terms of both intensity and lateralization. Finally, the interhemispheric correlation between beta power timecourses (extracted from the anatomically defined motor cortex parcels, *t*_0_ + 1 s: *t*_0_ + 2.5 s) revealed significantly higher correlation in both PLS patients (*P* = 0.0054) and ALS patients (*P* = 0.0086) relative to healthy controls. The implication of this finding is that beta power changes following completion of movement are abnormally coherent between hemispheres in these patient groups, but this may simply again reflect delayed PMBR. Beta rebound lateralization was unaffected in AGCs.

### Movement Inhibition

**Figure 6:**
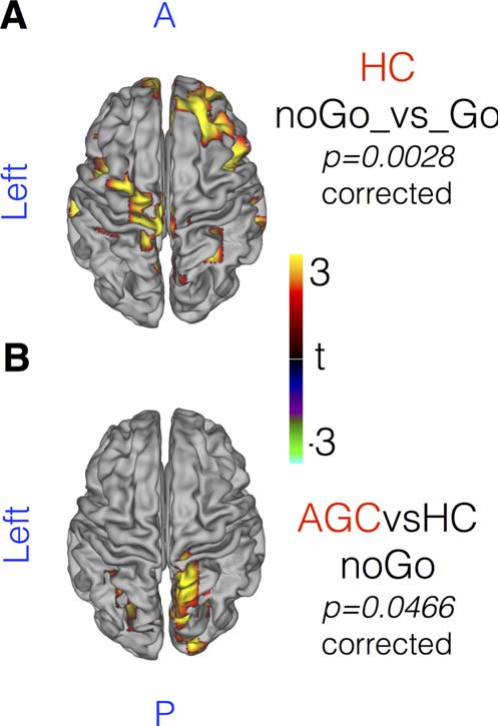
MEG data epoched around NoGo target presentation (*t* _0_ = target appearance). Beta‐band power increases over a diffuse cortical network in successfully inhibited NoGo trials, demonstrated in (**A)** healthy controls, beta power NoGo > Go. [*t*_0_ + 100 ms: *t*_0_ + 300 ms]. **B:** AGCs contrasted against HC, NoGo trials only, reveals increased beta power in posterior cortical regions. No significant differences are noted in ALS or PLS patients. *t*‐stats in ROY‐BIG‐BL. Whole brain cluster‐permutation correction. [Color figure can be viewed at http://wileyonlinelibrary.com.]

Available trials in which participants successfully withheld a preeminent motor response after appearance of the “NoGo” target were examined to probe integrity of the inhibitory cortical network. Faster responders made more false alarms on “NoGo” trials, as did those with deeper preparatory beta desychronization (*P* = 0.015). Contrasting correct “NoGo” trials with “Go” trials within the healthy control group, a relative increase in beta power was revealed in the frontal lobe and premotor regions. Data was re‐epoched around *t*_0_ = target presentation. The time period (*t*_0_ + 100 ms: *t*_0_ + 300 ms) was appraised in whole‐brain data, revealing a beta power difference (“NoGo” versus “Go” trials, healthy controls only) in clusters overlying the right frontal lobe and left motor cortex (*P* = 0.0028, Fig. 6A).

Group contrasts were restricted to successful “NoGo” trials during which the prepared response was inhibited. Within the same time‐period, no significant differences between ALS or PLS patients against controls were apparent in the degree to which beta power increased after “NoGo” targets. AGCs however demonstrated a relative increase in beta power in the right lateral occipital cortex, extending to the precuneus (*P* = 0.0466, Fig. 6B). Correlation analysis of beta power dynamics between selected cortical parcels (*t*_0_: *t*_0_ + 1 s) also suggested reduced functional connectivity between rIFC and SMA in PLS patients only (FWE *P* = 0.042). No group differences were noted in the correlation between motor cortex parcels, nor between ALS or AGC groups against controls.

### Clinical Correlations

Correlations between relevant behavioral measures, summary MEG statistics, and clinical metrics are presented in Table 2. These were exploratory given the small group numbers, and not corrected for multiple comparisons. Across all patients, slower responses were correlated with increasing ALSFRS‐R disability (global *P* = 0.005, upper limb *P* = 0.02), longer disease duration (*P* = 0.038) and higher UMN score (*P* = 0.019). Within the ALS group, impaired cognition (% correct ECAS or ACEr) tended to correlate with noGo mistakes (*P* = 0.073) as did UMN score (*P* = 0.057). Baselined PMBR correlated negatively with disease duration (*P* = 0.024) and positively with rate of progression (*P* = 0.036).

**Table 2:** Correlations within the ALS patients (Kendall's Tau, uncorrected) between clinical measures, behavioral data, and summary MEG statistics extracted from per‐subject ROI timecourses [Color table can be viewed at http://wileyonlinelibrary.com]

	ALSFRSr Sum	ALFRSr UL	UMN Score	Disease duration	Progression rate	Cognition	Median RT	noGo mistakes	Preparatory ERD	Execution ERD	ERD lateralization	PMBR
ALSFRSr Sum	x
ALFRSr UL	0.577*^*^	x
UMN Score	−0.333	−0.216	x
Disease Duration	0.187	−0.112	0.000	x
Progression Rate	−0.561*	−0.187	0.191	−0.552^**^	x
Cognition	0.374	0.299	−0.191	−0.139	0.127	x
Median RT	−0.524*	−0.785^**^	0.343	0.406*	0.164	−0.345	x
noGo Mistakes	−0.449	−0.299	0.458	−0.011	0.164	−0.418	0.418	x
Preparatory ERD	0.075	0.262	0.114	−0.177	−0.127	−0.345	−0.091	−0.018	x
Execution ERD	−0.224	0.000	0.381	−0.368*	−0.018	−0.309	0.164	0.236	0.673^**^	x
PMBR	−0.449	−0.150	0.000	0.014	0.491*	−0.091	0.091	0.091	−0.345	−0.236	0.345	X

a *P < 0.05, **P < 0.01.

## DISCUSSION

This study used MEG to investigate the neural basis of prepared voluntary movement generation, typified by modulation in beta‐band power, revealing abnormalities across the ALS phenotypic spectrum.

### Cortical Neurophysiological Dysfunction in ALS and PLS

Previous EEG investigations in ALS largely focused on evoked movement‐related cortical potentials (MRCPs), time‐locked to movement onset and averaged over many trials. The impact on these measures appears most prominent in patients with a high burden of UMN dysfunction [Bizovičar et al., 2013; Inuggi et al., 2011; Westphal et al., 1998], with MRCPs particularly reduced in patients with PLS [Bai et al., 2006]. Event‐related potentials (ERPs) have also revealed abnormal neural correlates of attention control [Pinkhardt et al., 2008], particularly within a bulbar‐onset sub‐group [Mannarelli et al., 2013]. This experimental design permitted assessment of neuronal function during a period of motor preparation, independent of and temporally distinct to subsequent motor performance, thus minimizing the potentially significant confound of group differences in motoric activity.

The precise physiological functions of beta oscillations and their task‐related modulations remain uncertain. More intense beta desychronization is induced as movements require more force [Stančák et al., 1997], speed [Toma et al., 2002], or complexity [Hummel et al., 2003; Manganotti et al., 1998]. Conversely, exaggerated and overly consistent beta synchrony is antikinetic, whether achieved though Parkinson's pathology [Little et al., 2012] or direct stimulation [Pogosyan et al., 2009]. Physiological beta synchrony is promoted in expectation of impending perturbation to a desired posture [Androulidakis et al., 2007] but is also sensitive to the uncertainty of motor outcome estimation [Tan et al., 2016]. Beta oscillations may also contribute to long‐range communication across cortical regions [Engel and Fries, 2010; Kopell et al., 2000] and can facilitate modulation of selective attention in support of action selection [Grent‐'t‐Jong et al., 2013, 2014; Tzagarakis et al., 2010], beyond simple correlation with reaction times [van Ede et al., 2012]. The abnormalities in this characteristic motor system rhythm displayed by ALS patients (amplified beta desychronization and attenuated beta rebound) may reflect or even contribute to an excitotoxic degeneration of neural microcircuitry, particularly given the apparent correlation with rate of disease progression. A more simplistic explanation of the beta desychronization difference in ALS patients might be the relative cognitive demands and task difficulty in comparison to healthy controls [Schoenfeld et al., 2005]. However, this fails to account for the corresponding abnormalities seen in high‐performing AGCs and the lack of excess beta desychronization within equally disabled PLS patients.

### Cortical Hyperexcitability in ALS

Although not unique to ALS [Di Lazzaro et al., 2004], converging strands of evidence suggest hyperexcitability as a key mechanism in the pathogenesis of ALS, possibly accompanied by a relative failure of inhibitory cortical interneuronal function [Turner and Kiernan, 2012]. Pathological studies demonstrate a particular vulnerability of parvalbumin‐positive interneurons [Maekawa et al., 2004; Nihei et al., 1993], later shown to contribute to reduced inhibitory GABA‐ergic tone in animal models [McGown et al., 2013; Nieto‐Gonzalez et al., 2011]. Neuroimaging studies across multiple modalities have supported the concept of reduced cortical inhibition. Initial observations were of a “boundary shift” in regional cerebral blood flow measured using positron emission tomography (PET) in ALS patients during a joystick task [Kew et al., 1993]. Further support stems from the observation of widened cortical BOLD activation in ALS patients during functional MRI (fMRI)‐based motor tasks [Mohammadi et al., 2011; Stanton et al., 2007b]; reduced binding of the PET GABA_A_ ligand [^11^C]‐fluamzenil [Lloyd et al., 2000; Turner et al., 2005a]; reduced GABA MR spectroscopy peak in the motor cortex [Foerster et al., 2012], and elevated Glx (glutamate and glutamine) peak in the medulla [Pioro et al., 1999].

Direct evidence of cortical hyperexcitability in ALS was provided by TMS [Vucic et al., 2013b]. Conditioning pulse protocols have reliably demonstrated reduced intracortical inhibition as a characteristic feature of ALS [Menon et al., 2015; Yokota et al., 1996; Ziemann et al., 1997], which may be partly ameliorated by reducing glutamatergic influence through administration of Riluzole—the only licensed disease‐modifying therapy in ALS [Stefan et al., 2001; Vucic et al., 2013a]. Postmovement beta rebound corresponds well to a period of reduced cortico‐spinal tract excitability, and the present data suggest that ALS patients differ in the speed of transition to this state after movement.

### The Endophenotype of PLS

The nosology of PLS continues to be debated [Le Forestier et al., 2001; Singer et al., 2007], although some clinical [Gordon et al., 2009; Pringle et al., 1992], neuroimaging [Agosta et al., 2014; Kolind et al., 2013; Kwan et al., 2013; Müller et al., 2012; Turner et al., 2007] and saccadic [Proudfoot et al., 2015] features support an apparent distinction from ALS. Involvement of the corpus callosum (CC) is a consistent feature of ALS [Filippini et al., 2010], thought to contribute to a functional impairment of interhemispheric inhibition, as evidenced by both TMS [Karandreas et al., 2007; Wittstock et al., 2007] and clinically evident mirror movements [Wittstock et al., 2011]. The CC appears to be a particularly vulnerable structure in PLS [Agosta et al., 2014; Ciccarelli et al., 2009; Iwata et al., 2011; Kolind et al., 2013] and a finding of delayed lateralization of PMBR is in keeping with this observation. Preserved hemispheric autonomy is still demonstrated by the time that PMBR peaks, suggesting still adequate CC functionality, although partial mirror movements during response epochs were also noted in some patients' EMG. Previous EEG‐based investigation of MRCPs also noted ipsilateral premotor recruitment among ALS patients with high UMN burden [Inuggi et al., 2011]. PLS patients by contrast demonstrated diminished preparatory MRCPs during a self‐paced EEG motor task [Bai et al., 2006]. These findings were not in this instance accompanied by any obvious alteration to MRCP topography and beta desychronization appeared preserved, highlighting that distinct neural generators underpin each phenomenon [Toro et al., 1994]. This study did not detect any significant excess in beta desychronization intensity between PLS patients and age‐matched controls in whole brain data. In contrast to the hyperexcitability typically detected in ALS, the motor cortex of PLS patients is often strikingly resistant to TMS stimulation [Brown et al., 1992; Kuipers‐Upmeijer et al., 2001; Zhai et al., 2003], in keeping with the present PLS data failing to demonstrate additional beta‐desychronization.

### Presymptomatic Cortical Dysfunction?

Characterization of any presymptomatic phase is a priority for all neurodegenerative conditions if preventative strategies are envisaged. Assessment of asymptomatic familial Alzheimer gene carriers has highlighted the added sensitivity of functional neuroimaging [Chhatwal et al., 2013] in keeping with the age‐dependent impact of APOE ε4 status on both fMRI [Filippini et al., 2011] and MEG [Cuesta et al., 2015] in the task‐free state. It remains an open question to what extent symptoms of ALS are preceded by temporally remote cellular abnormalities [Eisen et al., 2014]. Although animal models of ALS demonstrate abnormal neural architecture and function during embryonic stages [Martin et al., 2013; Vinsant et al., 2013], human epidemiological [Byrne et al., 2013; Schoder et al., 2010] and pathological [Proudfoot et al., 2014a] links to neurodevelopmental disorders remain sparse. Suggestions that ALS (or FTD) pathology might manifest in a behavioural prodrome long before diagnostic symptoms remain speculative [Eisen et al., 2014; Lule et al., 2008].

There are inconsistent reports of structural and functional MRI abnormalities prior to symptom onset in those at high genetic risk of ALS [Carew et al., 2011; Menke et al., 2016; Ng et al., 2008; Vucic et al., 2008; Walhout et al., 2015]. Evidence of cortical hyperexcitability in asymptomatic carriers of genetic mutations who are “at risk” of ALS is restricted to a very limited finding of reduced ligand [^11^C]‐flumazenil binding in two individuals with the D90A *SOD1* mutation [Turner et al., 2005a], and three *SOD1* mutation carriers who demonstrated reduced or absent intra‐cortical inhibition within three months of symptom onset [Vucic et al., 2008]. Further studies on seven asymptomatic *SOD1* mutation carriers (mean age 33 years) and 11 *C9orf72* carriers (mean age 49 years) failed to differentiate them from controls on TMS measures [Geevasinga et al., 2015a; Vucic et al., 2010]. The older age of the *SOD1* mutation carrier participants in the current study may contribute to the apparent gain in sensitivity offered by MEG.

Both patients with ALS and AGCs differ from controls in beta rebound latency, but the group effect directions are divergent. Review of the averaged EMG timecourses revealed that AGC participants completed their responses faster than controls, functionally corresponding to a more rapid transition from beta desychronization to beta rebound. However, across all groups EMG metrics and behavioral measures correlated poorly with beta rebound latency and intensity. Conflicting abnormalities between pre‐symptomatic carriers and manifest patients have previously been noted on fMRI studies of both Huntington's [Kloppel et al., 2009] and monogenetic Alzheimer's [Quiroz et al., 2010], therefore compensatory or pathological neural abnormalities may still underpin these MEG group differences. Slight differences in task performance pose a challenge in the interpretation of functional data from particularly motivated asymptomatic AGC. Blinded genetic testing of at‐risk individuals is a solution that raises novel ethical considerations as well as practical complexities, especially with regards to future therapeutic trials [Kim et al., 2015].

### Does Motoric Inhibition Reflect Executive Dysfunction?

Although cognitively impaired ALS patients are predictably under‐represented in demanding functional neuroimaging tasks, comparatively reduced activation in the dorsolateral prefrontal cortex has been demonstrated [Witiuk et al., 2014], while other frontal regions reveal increased activation during inhibition of prepared manual movements [Mohammadi et al., 2015]. The present finding of abnormal beta power in AGCs during successful inhibition was limited to posterior cortical regions in the context of task‐induced inferior and pre‐frontal activity. Despite previous findings of reduced stop‐signal ERPs [Thorns et al., 2010], no abnormalities in beta‐band dynamics were noted in ALS patients, so that the changes in AGCs might represent very early compensatory neural changes. In contrast to an ERP study utilising the Stroop task [Amato et al., 2013], but in keeping with eye‐tracking findings [Proudfoot et al., 2015], the present data suggest that executive control dysfunction may not spare PLS patients, given that task relevant functional connectivity was diminished between rIFC and SMA. Multiple nonexclusive biological and methodological restrictions may underlie the lack of correlation between the MEG data and cognitive profiles of the ALS participants [Verstraete et al., 2015], not least the brevity of the ECAS test as a opposed to more comprehensive and granular neuropsychological assessment.

### Study Limitations

These results complement existing EEG based investigations of sensorimotor oscillations in ALS which also found abnormally attenuated beta rebound but did not report increased beta desychronization [Bizovičar et al., 2014; Riva et al., 2012]. These previous studies differed from this investigation not only in acquisition technologies, but also in protocol design by requiring self‐paced movements of the right hand only, whereas the present study was a laterally cued task and thus facilitated analysis of a specific preparatory period. Additional analysis variables pertinent to neural signal interpretation include the selection of baseline period and frequency band of interest. Furthermore patient groups may differ in the degree to which they actively participate in a task, and this severely limits the conclusions that can be drawn from tasks of motor imagery (Kasahara et al., 2012; Lulé et al., 2007; Stanton et al., 2007a, 2007b). Riluzole administration could possibly dilute the reported ALS group effects, although analysis of the five remaining patients revealed a directionally similar trend. Unique challenges arise in the investigation of a genetically and phenotypically heterogenous condition such as ALS. Comparative studies against other neurological conditions with significant UMN burden of disease remain valuable. Furthermore, longitudinal study, particularly of a precisely defined group of AGCs before and after conversion to symptomatic ALS could pinpoint important aetiological pathways but might have restricted relevance to the wider sporadic ALS population.

MEG benefits from improved spatial resolution over EEG, as neuromagnetic signals pass through skull structures without spatial smearing. Reconstruction of the MEG signal into source‐space components overcomes the ambiguity inherent in selection of specific EEG sensors, but coregistration errors are still likely to limit precise anatomical conclusions regarding the current group differences. This methodology included selection of an ROI‐based purely on subject‐specific functional data, in addition to anatomically defined sources perhaps more vulnerable to coregistration error.

## CONCLUSIONS

Motor system cortical function assessed by beta‐band oscillations revealed abnormalities across the syndrome of ALS. MEG affords a useful contribution to the non‐invasive investigation of ALS pathology that complements more established techniques. The present results provide further distinguishing features between motor neurodegenerative phenotypes and supporting evidence of a detectable pre‐symptomatic phase to ALS pathophysiology. The current study has not exhausted the analytic options available from high‐dimensional MEG data, in particular further analysis of coherence measures may confirm and extend the existing literature concerning functional connectivity in ALS. Resting‐state networks similar in topography to those delineated by fMRI have also been decomposed from fluctuations in band‐limited MEG power [Brookes et al., 2011; Hipp et al., 2012] and the precise temporal sensitivity afforded by MEG has also enabled discovery of more rapidly cycling brain states [Baker et al., 2014] that remain unexplored across disease states. MEG was a well‐tolerated investigation for functionally disabled patients and could serve as a platform for the appraisal of novel therapeutic agents [Suntrup et al., 2013], as well as providing unique “real time” mechanistic insights into cortical dysfunction that will guide the emerging era of targeted therapeutics in ALS.

## Supporting information

Supporting Information Figure 1.

Supporting Information Figure 2.

Supporting Information Figure 3.

Supporting Information Figure 4.

Supporting Information Figure 5.
