# Supplementary figures and images for: Altered cortical beta‐band oscillations reflect motor system degeneration in amyotrophic lateral sclerosis
Source: Hum Brain Mapp. 2016 Sep 13;38(1):237–54. doi: 10.1002/hbm.23357 (PMC5215611; doi:10.1002/hbm.23357)

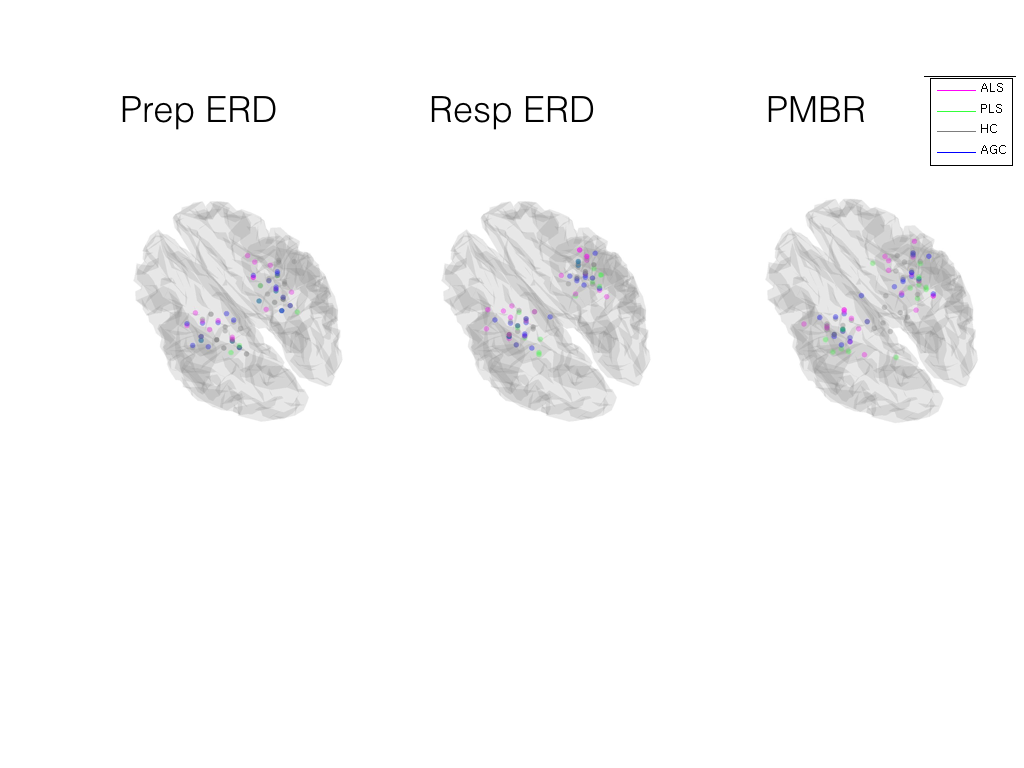

Supplement: Supplementary file 1 — Supporting Information Figure 1. [file HBM-38-237-s001.tiff]

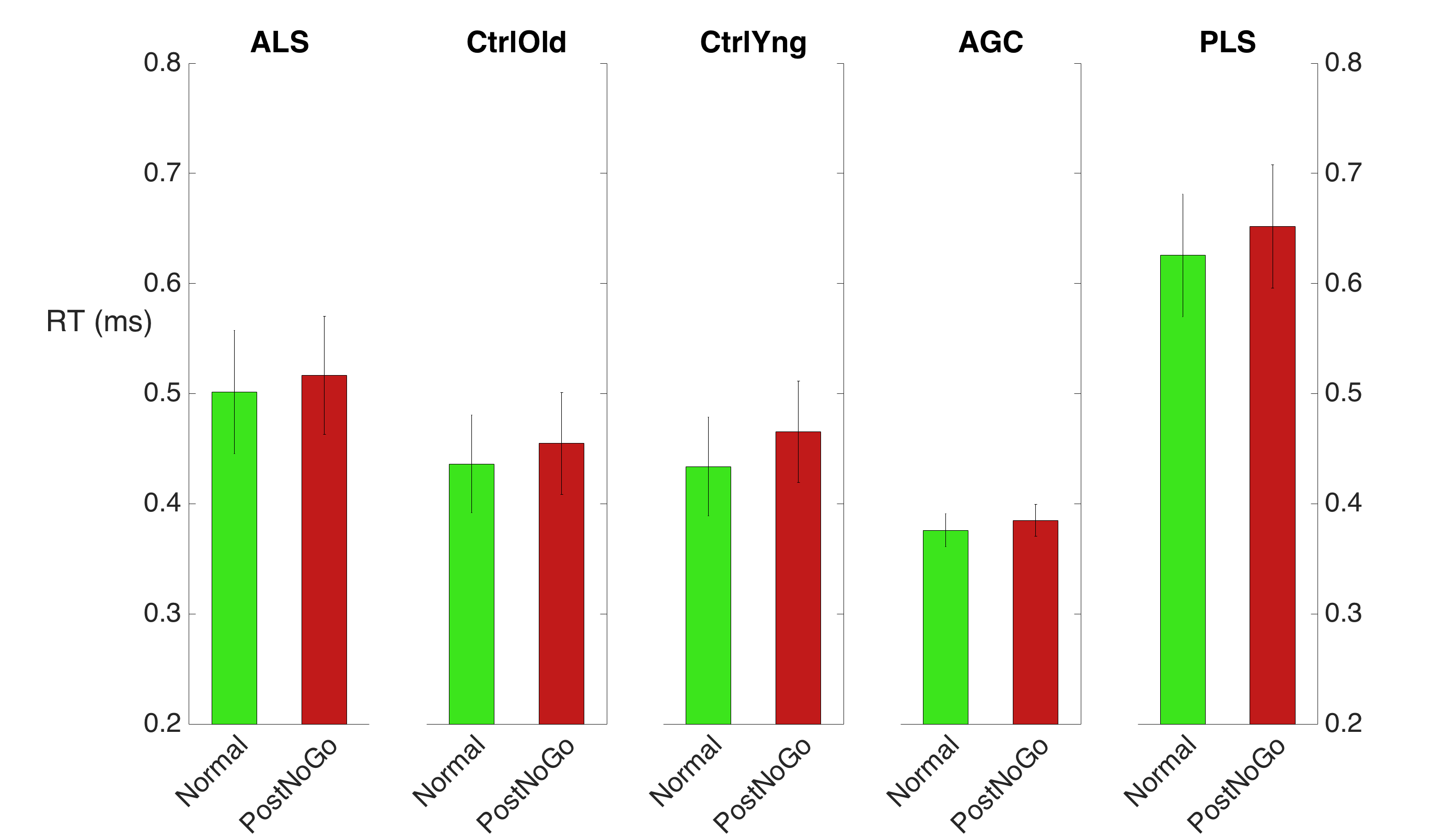

Supplement: Supplementary file 2 — Supporting Information Figure 2. [file HBM-38-237-s002.tif]

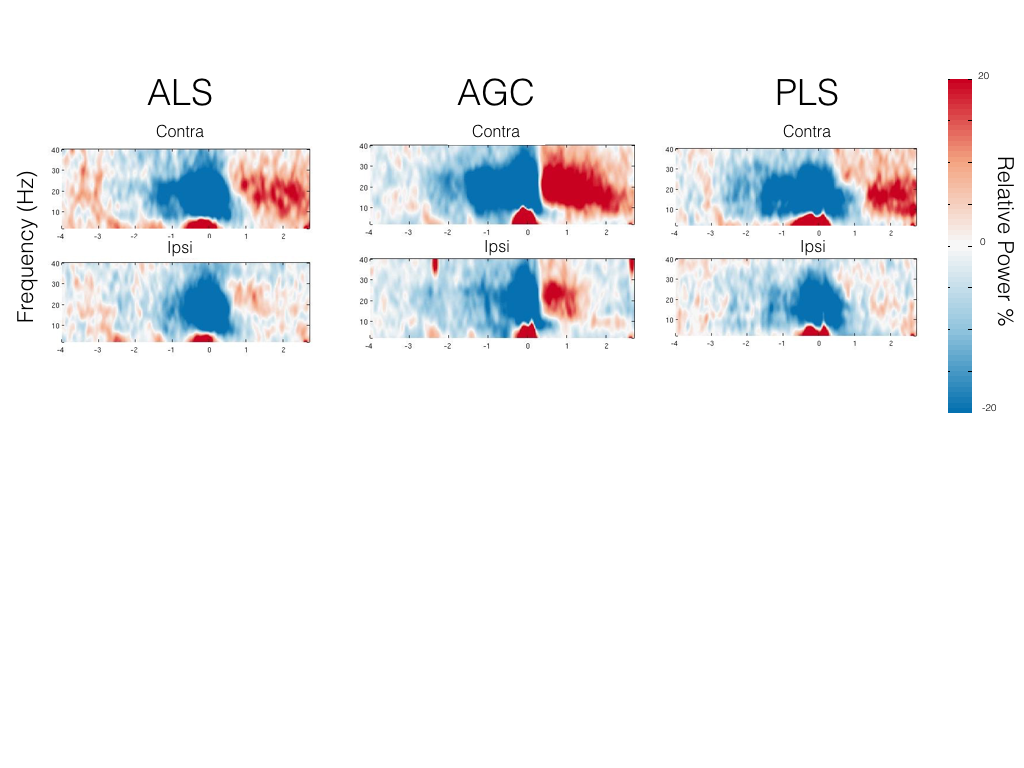

Supplement: Supplementary file 3 — Supporting Information Figure 3. [file HBM-38-237-s003.tiff]

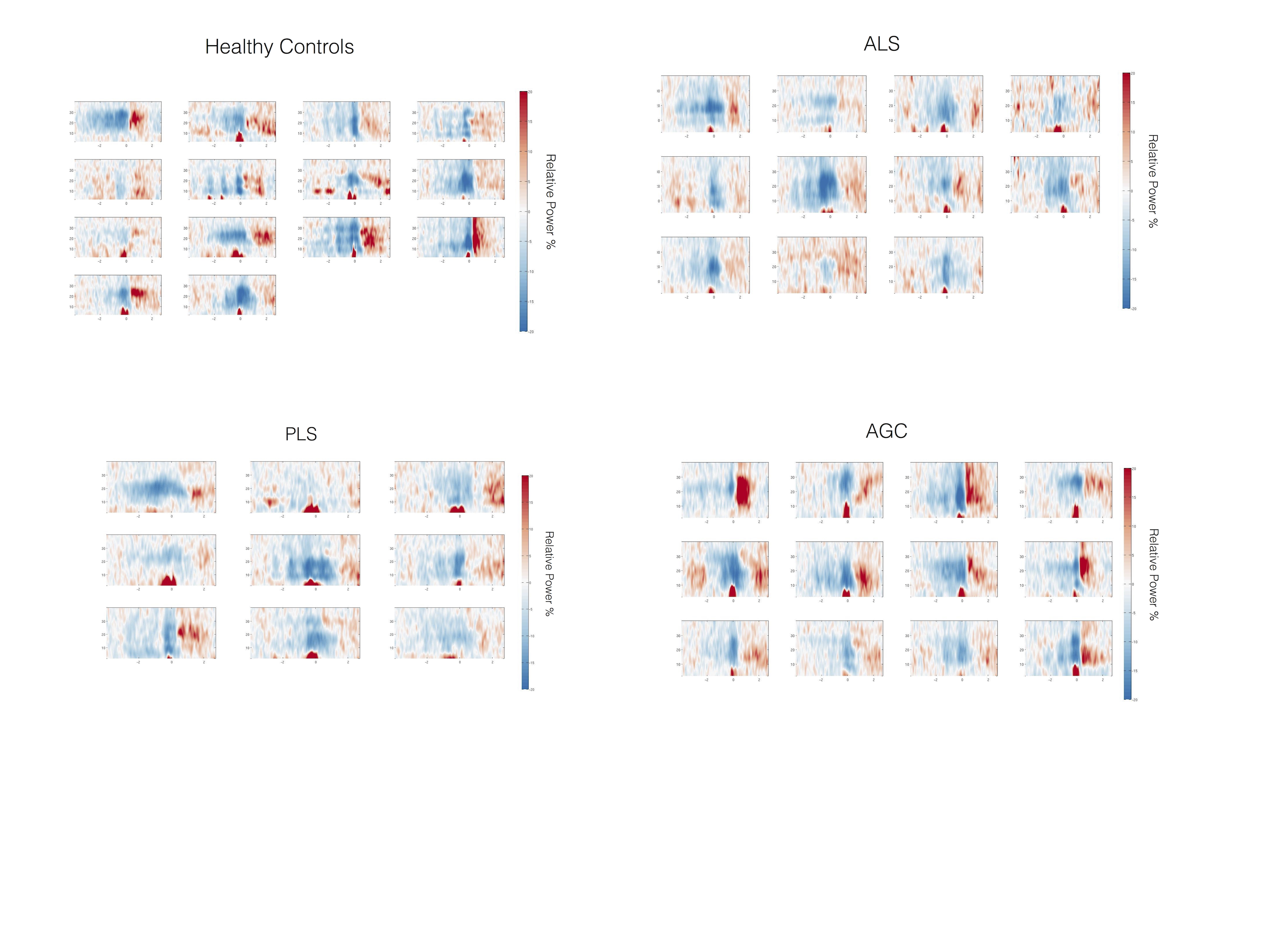

Supplement: Supplementary file 4 — Supporting Information Figure 4. [file HBM-38-237-s004.tiff]

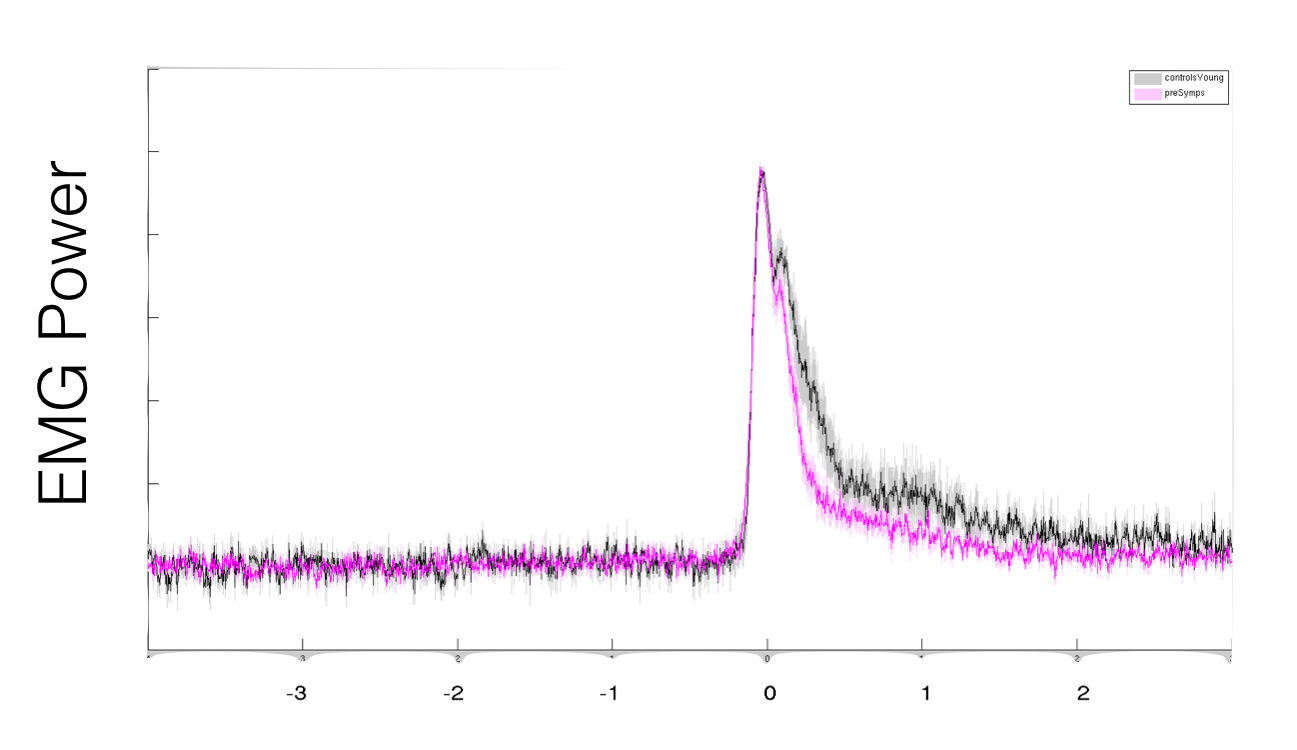

Supplement: Supplementary file 5 — Supporting Information Figure 5. [file HBM-38-237-s005.tiff]
